# Implant Survival in Patient Populations With a Mean Age of 65–75 Years Compared to Older Cohorts: A Systematic Review and Meta‐Analysis

**DOI:** 10.1111/clr.14456

**Published:** 2025-06-08

**Authors:** Samir Abou‐Ayash, Monika Bjelopavlovic, Pedro Molinero‐Mourelle, Martin Schimmel

**Affiliations:** ^1^ Department of Prosthetic Dentistry and Material Science University Medical Center of the Johannes Gutenberg University Mainz Mainz Germany; ^2^ Department of Reconstructive Dentistry and Gerodontology, School of Dental Medicine University of Bern Bern Switzerland

**Keywords:** advanced age, elderly patient, implant success, implant survival, meta‐analysis, systematic review

## Abstract

**Objectives:**

To evaluate implant survival and success rates in elderly patients, comparing younger old adults (65–75 years) to older implant patients (> 75 years).

**Methods:**

A systematic search was conducted using Medline, Cochrane Library, and PubMed Central for clinical studies on implant therapy in patients aged 65 and older. Outcomes included implant survival and success rates, peri‐implant parameters, bone‐level changes (BLC), and type of restoration and retention. Three‐ and five‐year survival and success rates, as well as implant loss per 100 implant‐years, were estimated with 95% confidence intervals (CI). Poisson regression models and incidence rate ratios (IRR) were used to compare study groups, and meta‐regression with restricted maximum likelihood estimation (REML) assessed BLC.

**Results:**

Twenty‐seven studies with a total of 3892 implants were included. Patients > 75 years had significantly higher five‐year survival rates (96.8%, CI: 95.9–97.5) compared to the 65–75 age group (92.1%, CI: 83.0–96.4; *p* = 0.031), with lower implant loss rates per 100 implant‐years. No significant difference in success rates was observed (*p* = 0.229). Although plaque and bleeding on probing (BOP) were more frequent in the older group, there was no significant difference in BLC (mean difference: 0.41 mm; *p* = 0.189). In patients > 65 with implant overdentures, single attachments showed significantly higher implant loss rates than bars (*p* = 0.035).

**Conclusions:**

Dental implants are a reliable treatment for older adults, including those over 75 years. Despite more frequent plaque and BOP in the older group, peri‐implant bone remained stable. Splinting implants in overdenture wearers aged > 65 is associated with 5.6 times higher survival rates.

## 1 Introduction

Dental implant therapy has become a cornerstone in reconstructive dentistry, providing a reliable solution for tooth replacement due to its high survival and success rates (Howe et al. 2019; Sailer et al. 2022; Roccuzzo et al. 2022). At the same time, an increase in median age has been observed in many regions of the world (Rudnicka et al. 2020). With increasing age, however, the likelihood of tooth loss also increases (Kanasi et al. 2016), and therefore implant therapy is becoming more and more of a focus also in older patients (Schimmel et al. 2018). Exemplary data from Switzerland shows that 26.5% of a representative population sample older than 65 years of age already have implant prostheses, with a still increasing trend over the last 20 years (BFS 2025). However, the success and longevity of dental implants in this demographic remain a subject of ongoing research and debate.

Aging is usually accompanied by physiological changes that could potentially affect the osseointegration and long‐term stability of dental implants (Kanasi et al. 2016). Factors such as decreased bone density, changes in the immune response, and a higher prevalence of systemic conditions might contribute to an elevated risk of implant failure, complications, and increased bone resorption around implants in older adults (Bornstein et al. 2009; Feher et al. 2020; Jung et al. 2023). Previous studies have reported mixed results, with some indicating a higher failure and/or complication rates in older patients (Howe et al. 2019; Porter and Von Fraunhofer 2005), while others suggest comparable outcomes across different age groups (Schimmel et al. 2018; Schenk et al. 2024). However, no clear age threshold has been established to differentiate implant outcomes in older adults. Given the increasing number of elderly individuals receiving dental implants, further research is necessary to determine whether age alone is a significant risk factor.

The present systematic review seeks to summarize the current evidence on implant survival rates in older adults, comparing implant survival rates in patients between 65 and 75 years to survival rates in patients older than 75 years. Furthermore, implant success, the presence of plaque, bleeding on probing (BOP), the rate of peri‐implant bone‐level alterations (MBL), and the effects of the jaw (maxilla vs. mandible), restoration type, and the retention type (in implant overdentures) on implant survival were analyzed.

## 2 Material and Methods

### 2.1 Study Protocol and Search Strategy

The present systematic literature review was conducted according to the PRISMA guidelines (Preferred Reporting Items for Systematic Reviews and Meta‐Analyses) (Moher et al. 2009). The protocol for the literature review was registered in the “International Prospective Register of Systematic Reviews” (PROSPERO) under the number CRD42023478900. The primary research question for the literature review was defined following the PICO model:

(P)opulation: patients who received dental implants aged 65 or above.

(I)ntervention: 65–75 years group.

(C)omparison: > 75 years group (O)utcome: Implant survival rates.

PICO Question: Are there differences in implant survival and success rates between patients aged 65 to 75 years and those older than 75 years?

The systematic literature review was conducted without applying additional filters in the following online databases:

- Medline (PubMed) (incl. Epub Ahead of Print, In‐Process & Other Non‐Indexed Citations) (Timeframe: 1946–20.10.2023)
- Cochrane Library (Wiley) (Timeframe: 1996–20.10.2023)
- PubMed Central (1946–20.10.2023)

The search algorithm was defined based on studies included in a previous systematic review (Schimmel et al. 2018). This search term was developed using Medical Subject Headings (MeSH) as well as the titles, abstracts, and keywords of the studies included in the aforementioned systematic review. Initially, a test search algorithm was developed, which included additional relevant technical vocabulary identified using the web‐based software “Yale MeSH Analyzer” and “PubReMiner.” This test algorithm was then used for an initial search run in Medline to check whether all studies from the previous systematic review appeared in the current search. The search algorithm was subsequently modified until all studies could be identified using the algorithm. The final search algorithms are reported in Appendix 1. The searches were conducted without additional search filters, and the search results were subsequently imported into the web‐based software Rayyan. There, duplicates were identified by the software, followed by the manual study selection based on the inclusion and exclusion criteria began.

### 2.2 Study Selection and Data Extraction Process

The study selection was carried out independently by two researchers (M.B. and P.‐M.‐M.) based on the following inclusion and exclusion criteria:

#### 2.2.1 Inclusion Criteria

- Human studies
- Average age of the relevant study cohort at least 65 years
- At least 10 patients per relevant cohort
- Placement of at least one dental implant/patient
- Publications in German or English

#### 2.2.2 Exclusion Criteria

- In vitro or animal studies
- Study cohorts with an average age < 65 years
- Publications in languages other than German or English

For calibration purposes, the two involved researchers analyzed the first 20 titles together and jointly decided whether the study should be included or not. Afterwards, the researchers started with the independent screening process. The study selection was carried out step‐by‐step, initially based on the study titles, followed by abstract screening, and finally the full texts. If, for example, it was unclear based on the title whether the inclusion criteria were met, the study was included in the next selection step for better assessment. After each step, the two investigators compared the results of their individual selection process and involved a third person (S.A.‐A.) for decision‐making in case of disagreement about the eligibility of a study. Cohen's kappa score was used, assessing the degree of agreement between the reviewers' individual assessments. After the full‐text screening, M.B. and P.‐M.‐M. performed the data extraction individually, using a data extraction sheet with the following variables: author, study design, mean patient age, jaw (upper/lower), number of implants at baseline and follow‐up, type of restoration, number of implants per reconstruction, type of retention follow‐up, implant survival, implant success, and marginal bone level alterations, prosthetic complications, BOP, and presence of plaque. If multiple relevant outcomes were analyzed in one study, all outcome data were extracted individually. When data from the same cohort were reported at multiple follow‐up time points within a study or in consecutive studies, only information from the most recent report were extracted. If data could not be extracted due to missing information, the corresponding authors were contacted to obtain additional information. If the corresponding authors did not reply or could not provide the requested data, studies were excluded from further evaluation, and the reason for exclusion was noted.

### 2.3 Risk of Bias

The two researchers independently assessed the risk of bias of included studies, using either the Cochrane Risk of Bias tool (RoB 2.0) for randomized trials (Sterne et al. 2019) and the Risk of Bias in Non‐Randomized Studies tool (ROBINS‐I) in the case of non‐randomized trials (Sterne et al. 2016), and compared their individual assessments afterward. The results of the risk of bias analysis were visualized using the risk of bias.

### 2.4 Statistical Analysis

The analyses of survival and success were conducted under the assumption of Poisson‐distributed events. In each study, the exposure time was calculated as the cumulative time, derived from the number of implants at baseline and the mean follow‐up time of the study. For the individual studies, survival and success rates with 95% confidence intervals (95% CI) were estimated for 3–5 years. Additionally, the estimation of loss and failure rates with 95% CI was conducted for a 100‐year exposure. For studies without events, one‐sided confidence intervals were calculated.

The estimation and comparison of survival and success rates, as well as the estimation of event rates per 100 years in subgroups (i.e., age < 75), were performed either using a random‐effects Poisson regression (for subgroups with more than one study) or a Poisson regression (for subgroups with one study). For variables with exactly two categories, incidence rate ratios were additionally estimated. For categories with at least two or more studies, the variance of exponentiated random effects was calculated to assess heterogeneity. The same approach was used to evaluate BOP and Plaque positive rates. However, in this case, only the number of implants per study was considered, not the mean follow‐up (FU) time. Whether the two age groups differ in terms of mean MBLs was analyzed using a meta‐regression with REML estimation. Stata/IC 16.0 for Unix was used for statistical analysis. All statistical tests are two‐sided at a significance level of 0.05.

Estimated implant survival and success rates after 3 years and 5 years, and implant loss, and failure rates per 100 years were estimated by Poisson regression. Assuming that the total number of events follows a Poisson distribution, a regression model was used to model the rate of random events that occur in the exposure time, for example, the complication rate over a fixed period of time. Furthermore, survival rates with 95% confidence intervals after 3 and 5 years were calculated using the relationship between event rate and survival function (*S*) (*S*(*T*) = exp.(−*T* × event rate)). In addition, Poisson regression was used to estimate the rate at which events occurred (incidence rate) within subgroups and to compare the incidence rates of subgroups by calculating incidence rate ratios (IRRs) with 95% confidence intervals (CI). All statistical tests were two‐sided (*α* = 0.05). Stata/IC 16.0 for Windows (StataCorp LLC, 4905 Lakeway Drive, College Station, TX 77,845, USA) was used for statistical analysis.

## 3 Results

**FIGURE 1:**
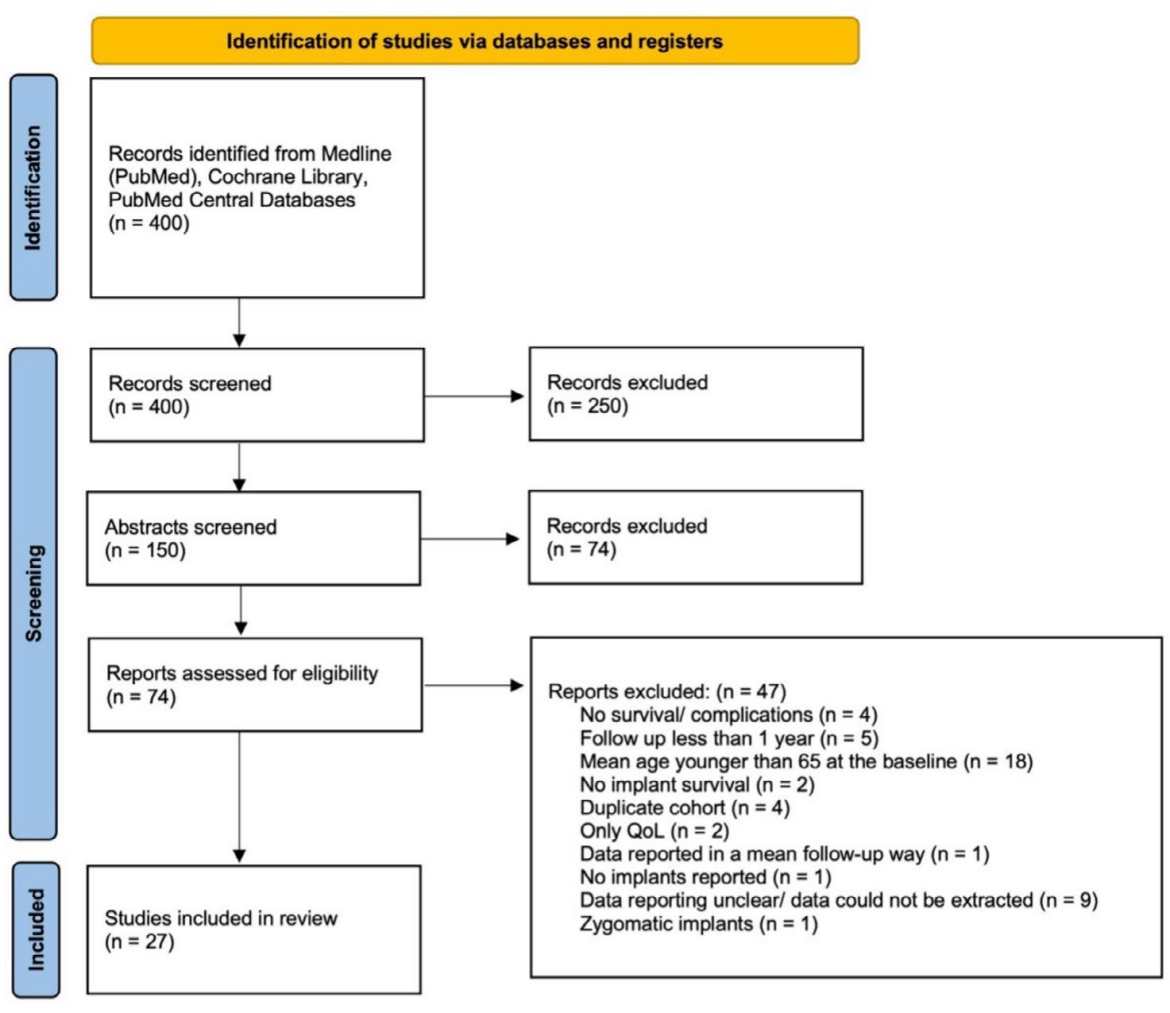
PRISMA flow‐diagram.

Initially, 400 records were identified during the systematic literature search and were screened by title after duplicate elimination. After a consensus was reached for the title screening, 150 abstracts were screened. After further consensus, 74 full texts were analyzed, of which 27 were included for data extraction (Figure 1). The kappa scores were 0.97 for the title screening, 0.85 for the abstract screening, and 0.87 for the full‐text screening (very good agreement). Data could finally be extracted from 27 studies. The reasons for study exclusion at the data‐extraction stage are provided in Appendix 2.

### 3.1 Description of Included Studies

Among the 27 included studies, 8 studies were RCTs (Al‐Nawas et al. 2012; Alsabeeha et al. 2011; Enkling et al. 2022; Giannakopoulos et al. 2017; Gothberg et al. 2014; Ma et al. 2010; Maniewicz et al. 2019; Schuster et al. 2020), 17 were prospective studies (Bakker et al. 2019; Becker et al. 2016; Bryant and Zarb 2003; Chow et al. 2017; Enkling et al. 2020; Harder et al. 2011; Hoeksema et al. 2016; Huber et al. 2012; Jemt 1993; Khoo et al. 2013; Leventi et al. 2014; Morneburg and Proschel 2008; Mericske‐Stern and Zarb 1993; Vervaeke et al. 2018; Rammelsberg et al. 2014; Di Torresanto et al. 2014; Zhang et al. 2019) and 2 were retrospective studies (Friberg and Jemt 2015; Rentsch‐Kollar et al. 2010). Although there were 8 RCTs, none of the studies compared the impact of patient age on implant survival.

The total number of implants that were followed up was *n* = 3892. Among those implants, 811 implants were placed in patients older than 75 years, and 3081 implants were placed in patients aged 65–75 years. Most of the implants were used to support/retain overdentures (*n* = 1913), followed by fixed restorations (*n* = 1374), mixed restorations (*n* = 518), and implant‐assisted removable partial dentures (*n* = 87). The follow‐up time ranged from 1 to 20 years, with an average follow‐up of 4.4 years. The main study characteristics are presented in Table 1, including the estimated 3 and 5 year implant survival, and the implant loss rate per 100 implant‐years. A total of 16 studies, including 20 relevant study cohorts, focused on implant success (Table 2). Success criteria varied within those studies: Five studies applied the success criteria described by Buser et al. (1990), four studies defined success as complication‐free survival, one study defined success by the maximum acceptable peri‐implant bone loss of 1 mm, one study described implants as successful when they showed clinical stability, full functionality, no pain on light percussion on the implant, peri‐implant soft tissue health, and no radiolucency around the implants or other radiographic indications of pathology. Five studies reported implant success without a description of the success criteria.

**TABLE 1:** Description of included studies on implant survival.

Study	Age	Jaw		Retention	#Impl.at FU	Mean FU [y]	Total FU [y]	#Losses	#Survival	Survival rate (95% CI)*		Losses per 100 implants (95% CI)*
			Type ofrestor.							3 year	5 year	
Al Nawas et al. (2012)	≤ 75	Mandible	OD	Single	82	1.0	82	1	81	96.4 (81.0–99.9)	94.0 (70.3–99.8)	1.22 (0.03–6.79)
Al Nawas et al. (2012)	≤ 75	Mandible	OD	Single	78	1.0	78	2	76	92.5 (74.7–99.1)	87.8 (61.5–98.5)	2.56 (0.31–9.26)
Alsabeeha et al. (2011)	≤ 75	Mandible	OD	Single	12	1.0	12	3	9	42.2 (2.0–85.3)	23.7 (0.1–76.8)	25.00 (5.16–73.06)
Alsabeeha et al. (2011)	≤ 75	Mandible	OD	Single	12	1.0	12	0	12	100 (33.2–100)	100 (15.9–100)	0 (0–30.74)
Alsabeeha et al. (2011)	≤ 75	Mandible	OD	Single	12	1.0	12	0	12	100 (33.2–100)	100 (15.9–100)	0 (0–30.74)
Bakker et al. (2019)	> 75	Mandible	OD	Splinted	30	20.0	600	2	28	99.0 (96.4–99.9)	98.3 (94.1–99.8)	0.33 (0.04–1.20)
Becker et al. (2016)	> 75	Both	Mixed		83	1.0	83	1	82	96.4 (81.2–99.9)	94.1 (70.7–99.8)	1.20 (0.03–6.71)
Bryant and Zarb (2003)	≤ 75	Both	Mixed		132	10.0	1320	10	122	97.7 (95.9–98.9)	96.3 (93.2–98.2)	0.76 (0.36–1.39)
Chow et al. (2017)	> 75	Mandible	OD	Single	126	5.2	660.2	2	124	99.1 (96.8–99.9)	98.5 (94.7–99.8)	0.30 (0.04–1.09)
Enkling et al. (2020)	≤ 75	Mandible	OD	Single	76	5.0	380	0	76	100 (97.1–100)	100 (95.2–100)	0 (0–0.97)
Enkling et al. (2022)	≤ 75	Mandible	OD	Splinted	58	3.0	174	0	58	100 (93.8–100)	100 (89.8–100)	0 (0–2.12)
Friberg and Jemt (2015)	≤ 75	Mandible	Fixed		273	5.0	1365	1	272	99.8 (98.8–100)	99.6 (98.0–100)	0.07 (0–0.41)
Friberg and Jemt (2015)	≤ 75	Mandible	Fixed		512	5.0	2560	15	497	98.3 (97.1–99.0)	97.1 (95.3–98.4)	0.59 (0.33–0.97)
Friberg and Jemt (2015)	≤ 75	Mandible	Fixed		245	5.0	1225	4	241	99.0 (97.5–99.7)	98.4 (95.9–99.6)	0.33 (0.09–0.84)
Friberg and Jemt (2015)	≤ 75	Mandible	Fixed		200	5.0	1000	3	197	99.1 (97.4–99.8)	98.5 (95.7–99.7)	0.30 (0.06–0.88)
Giannakopoulos et al. (2017)	≤ 75	Mandible	OD	Single	92	1.0	92	1	91	96.8 (82.9–99.9)	94.7 (73.2–99.9)	1.09 (0.03–6.06)
Gothberg et al. (2014)	≤ 75	Both	Fixed		144	1.0	144	4	140	91.9 (80.1–97.8)	86.9 (69.2–96.3)	2.78 (0.76–7.11)
Harder et al. (2011)	≤ 75	Mandible	OD	Single	11	3.6	39.6	0	11	100 (74.6–100)	100 (61.3–100)	0 (0–9.32)
Hoeksema et al. (2016)	≤ 75	Mandible	OD	Splinted	68	10.0	680	4	64	98.2 (95.6–99.5)	97.1 (92.7–99.2)	0.59 (0.16–1.51)
Huber et al. (2012)	≤ 75	Both	Mixed		65	1.0	65	0	65	100 (83.9–100)	100 (74.7–100)	0 (0–5.68)
Jemt (1993)	> 75	Both	Mixed		238	3.0	714	7	231	97.1 (94.1–98.8)	95.2 (90.3–98.0)	0.98 (0.39–2.02)
Khoo et al. (2013)	> 75	Mandible	OD	Splinted	86	1.0	86	0	86	100 (87.7–100)	100 (80.3–100)	0 (0–4.29)
Leventi et al. (2014)	≤ 75	Both	OD	Unclear	102	7.7	785.4	1	101	99.6 (97.9–100)	99.4 (96.5–100)	0.13 (0–0.71)
Maniewicz et al. (2019)	> 75	Mandible	OD	Splinted	32	2.7	86.4	0	32	100 (87.7–100)	100 (80.4–100)	0 (0–4.27)
Mericske‐Stern and Zarb (1993)	≤ 75	Mandible	OD	Splinted	63	5.0	315	5	58	95.3 (89.3–98.5)	92.3 (82.8–97.4)	1.59 (0.52–3.70)
Mericske‐Stern and Zarb (1993)	≤ 75	Mandible	OD	Mixed	68	5.0	340	6	62	94.8 (88.9–98.1)	91.5 (82.2–96.8)	1.76 (0.65–3.84)
Morneburg and Proschel (2008)	≤ 75	Mandible	OD	Single	128	6.0	768	6	122	97.7 (95.0–99.1)	96.2 (91.8–98.6)	0.78 (0.29–1.70)
Rammelsberg et al. (2014)	≤ 75	Both	OD	Unclear	141	2.7	380.7	21	120	84.3 (76.8–90.1)	75.3 (64.4–84.1)	5.52 (3.41–8.43)
Rammelsberg et al. (2014)	≤ 75	Both	RP		87	2.7	234.9	7	80	91.3 (82.7–96.4)	86.0 (72.8–94.2)	2.98 (1.20–6.14)
Rentsch‐Kollar et al. (2010)	> 75	Mandible	OD	Splinted	216	16.5	3564	26	190	97.8 (96.8–98.6)	96.4 (94.8–97.6)	0.73 (0.48–1.07)
Schuster et al. (2020)	≤ 75	Mandible	OD	Single	20	1.0	20	3	17	61.4 (17.7–91.0)	44.4 (5.6–85.5)	15.00 (3.09–43.84)
Schuster et al. (2020)	≤ 75	Mandible	OD	Single	20	1.0	20	2	18	72.9 (26.1–96.4)	59.0 (10.6–94.1)	10.00 (1.21–36.12)
Ma et al. (2010)	≤ 75	Mandible	OD	Splinted	158	10.0	1580	0	158	100 (99.3–100)	100 (98.8–100)	0 (0–0.23)
Di Torresanto et al. (2014)	≤ 75	Mandible	OD	Single	40	2.0	80	0	40	100 (86.8–100)	100 (79.0–100)	0 (0–4.61)
Vervaeke et al. (2018)	≤ 75	Mandible	OD	Single	48	2.0	96	0	48	100 (88.9–100)	100 (82.2–100)	0 (0–3.84)
Zhang et al. (2019)	≤ 75	Mandible	OD	Single	134	5.9	790.6	2	132	99.2 (97.3–99.9)	98.7 (95.5–99.8)	0.25 (0.03–0.91)

Abbreviations: FC, fixed complete; FU, follow‐up; OD, overdenture; RP, removable partial.

* Assuming poisson distributed failures of implants.

**TABLE 2:** Description of included studies on implant success.

Study	Age	Jaw	Type of restor.	Retention	#Impl.at FU	Mean FU [y]	Total FU [y]	#Failures	#Success	Estimated success rate (95% CI)		Estimated failure rate per 100 implants (95% CI)*
										3 year	5 year	
Al Nawas et al. (20122)	≤ 75	Mandible	OD	Single	82	1.0	82	3	79	89.4 (71.2–97.8)	83.0 (56.8–96.3)	3.66 (0.75–10.69)
Al Nawas et al. (2012)	≤ 75	Mandible	OD	Single	78	1.0	78	4	74	85.4 (65.6–95.9)	76.9 (49.5–93.2)	5.13 (1.40–13.13)
Alsabeeha et al. (2011)	≤ 75	Mandible	OD	Single	12	1.0	12	3	9	42.2 (2.0–85.3)	23.7 (0.1–76.8)	25.00 (5.16–73.06)
Alsabeeha et al. (2011)	≤ 75	Mandible	OD	Single	12	1.0	12	0	12	100 (33.2–100)	100 (15.9–100)	0 (0–30.74)
Alsabeeha et al. (2011)	≤ 75	Mandible	OD	Single	12	1.0	12	0	12	100 (33.2–100)	100 (15.9–100)	0 (0–30.74)
Bakker et al. (2019)	> 75	Mandible	OD	Splinted	30	20.0	600	2	28	99.0 (96.4–99.9)	98.3 (94.1–99.8)	0.33 (0.04–1.20)
Becker et al. (2016)	> 75	Both	Mixed		83	1.0	83	1	82	96.4 (81.2–99.9)	94.1 (70.7–99.8)	1.20 (0.03–6.71)
Enkling et al. (2020)	≤ 75	Mandible	OD	Single	76	5.0	380	0	76	100 (97.1–100)	100 (95.2–100)	0 (0–0.97)
Enkling et al. (2022)	≤ 75	Mandible	OD	Splinted	58	3.0	174	3	55	94.9 (85.6–98.9)	91.7 (77.2–98.2)	1.72 (0.36–5.04)
Giannakopoulos et al. (2017)	≤ 75	Mandible	OD	Single	92	1.0	92	1	91	96.8 (82.9–99.9)	94.7 (73.2–99.9)	1.09 (0.03–6.06)
Gothberg et al. (2014)	≤ 75	Both	FC		144	1.0	144	4	140	91.9 (80.1–97.8)	86.9 (69.2–96.3)	2.78 (0.76–7.11)
Harder et al. (2011)	≤ 75	Mandible	OD	Single	11	3.6	39.6	0	11	100 (74.6–100)	100 (61.3–100)	0 (0–9.32)
Huber et al. (2012)	≤ 75	Both	Mixed		65	1.0	65	0	65	100 (83.9–100)	100 (74.7–100)	0 (0–5.68)
Leventi et al. (2014)	≤ 75		OD		102	7.7	785.4	5	97	98.1 (95.6–99.4)	96.9 (92.8–99.0)	0.64 (0.21–1.49)
Mericske‐Stern and Zarb (1993)	≤ 75	Mandible	OD	Splinted	63	5.0	315	6	57	94.4 (88.1–97.9)	90.8 (80.9–96.6)	1.90 (0.70–4.15)
Mericske‐Stern and Zarb (1993)	≤ 75	Mandible	OD	Mixed	68	5.0	340	5	63	95.7 (90.1–98.6)	92.9 (84.0–97.6)	1.47 (0.48–3.43)
Rentsch‐Kollar et al. (2010)	> 75	Mandible	OD	Splinted	216	16.5	3564	43	173	96.4 (95.2–97.4)	94.1 (92.1–95.7)	1.21 (0.87–1.63)
Ma et al. (2010)	≤ 75	Mandible	OD	Splinted	158	10.0	1580	16	142	97.0 (95.2–98.3)	95.0 (92.0–97.1)	1.01 (0.58–1.64)
Di Torresanto et al. (2014)	≤ 75	Mandible	OD	Single	40	2.0	80	0	40	100 (86.8–100)	100 (79.0–100)	0 (0–4.61)
Vervaeke et al. (2018)	≤ 75	Mandible	OD	Single	48	2.0	96	0	48	100 (88.9–100)	100 (82.2–100)	0 (0–3.84)

Abbreviations: FC, fixed complete; FU, follow‐up; OD, overdenture; RP, removable partial.

### 3.2 Risk of Bias Assessment

**FIGURE 2:**
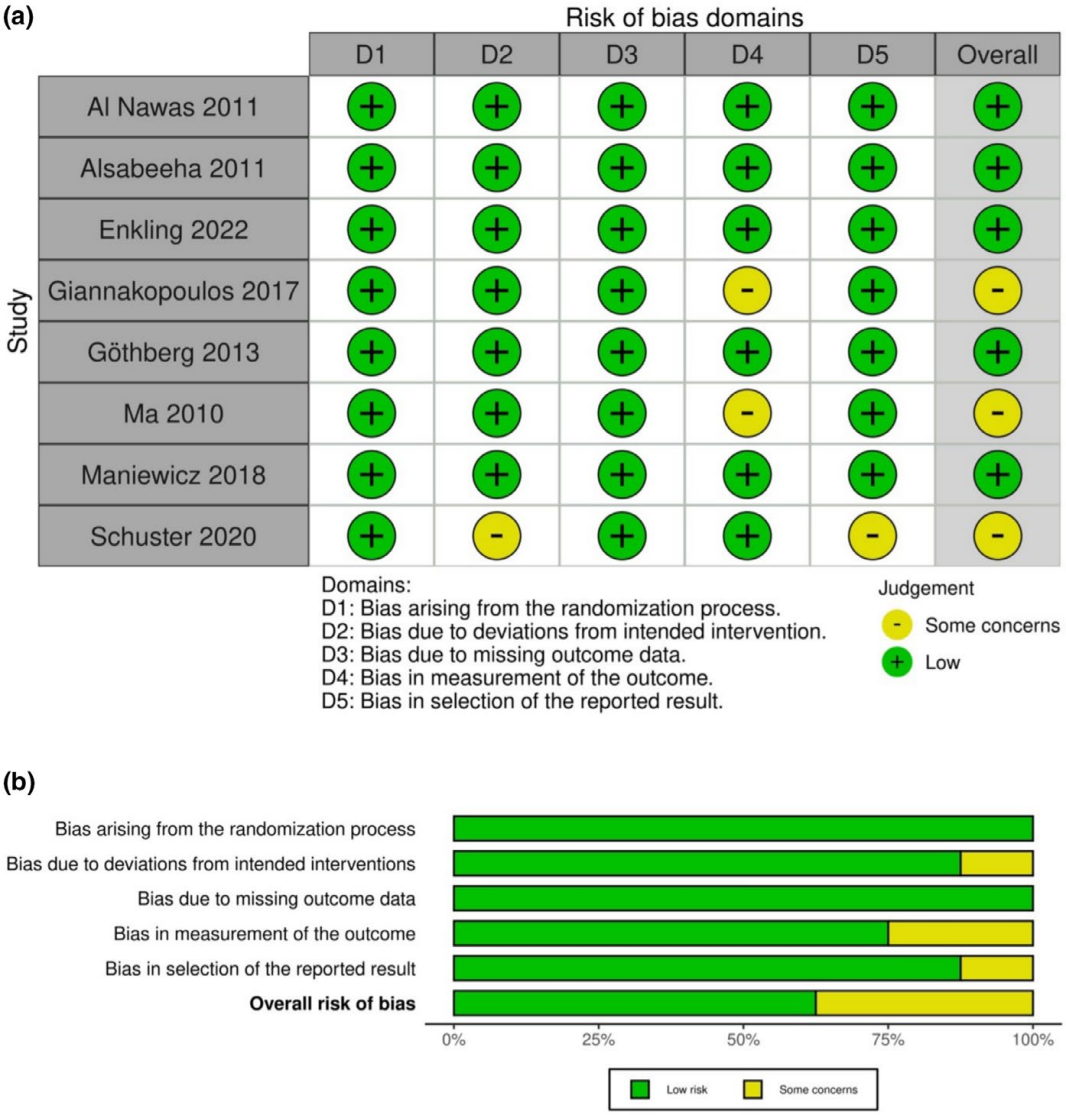
Risk of bias analysis of included randomized controlled clinical studies.

The risk of bias analyses of included RCTs showed 5 studies with “low” risk of bias (Al‐Nawas et al. 2012; Alsabeeha et al. 2011; Enkling et al. 2022; Gothberg et al. 2014; Maniewicz et al. 2019) and three with “some concerns” (Giannakopoulos et al. 2017; Schuster et al. 2020; Ma et al. 2010) (Figure 2a,b).

**FIGURE 3:**
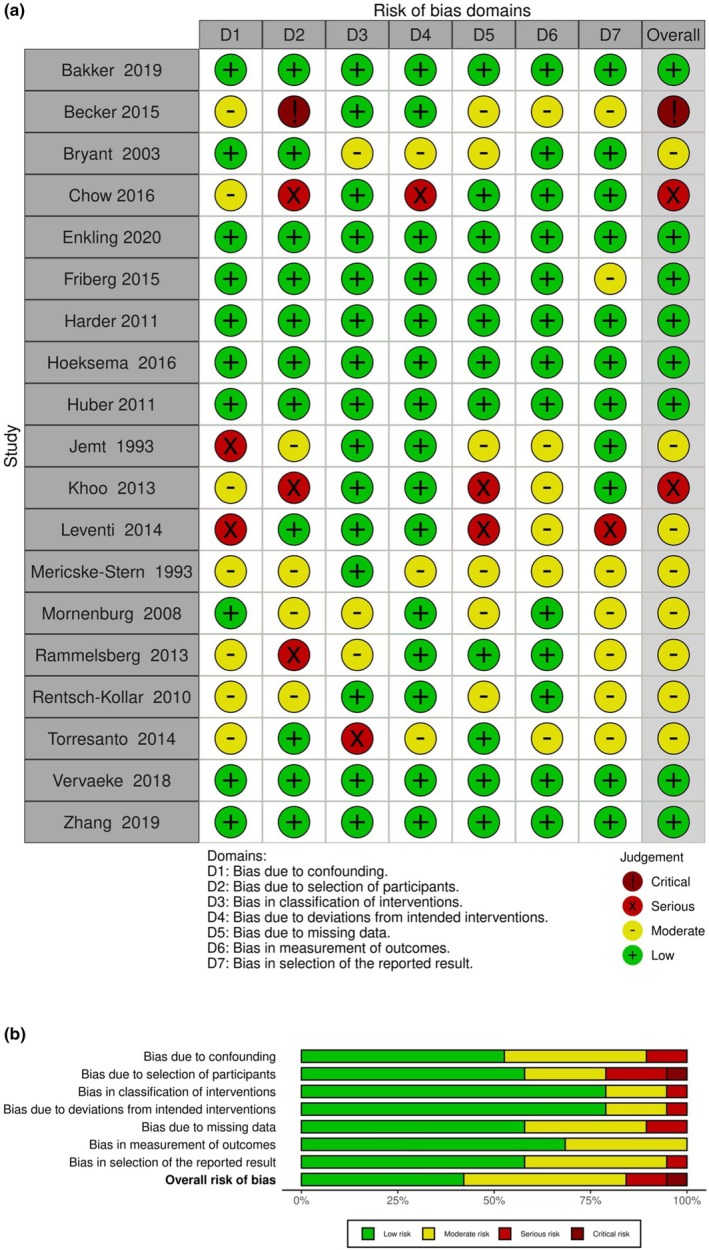
Risk of bias analysis of included non‐randomized studies.

The analyses of the non‐randomized studies showed eight out of 19 reports with “low” risk of bias (Bakker et al. 2019; Enkling et al. 2020; Friberg and Jemt 2015; Harder et al. 2011; Hoeksema et al. 2016; Huber et al. 2012; Vervaeke et al. 2018; Zhang et al. 2019), eight with “moderate” risk (Bryant and Zarb 2003; Jemt 1993; Leventi et al. 2014; Mericske‐Stern and Zarb 1993; Morneburg and Proschel 2008; Rammelsberg et al. 2014; Rentsch‐Kollar et al. 2010; Di Torresanto et al. 2014), two had “serious” risk (Chow et al. 2017; Khoo et al. 2013) and one critical risk of bias (Becker et al. 2016) (Figure 3a,b).

## 4 Meta Analyses

### 4.1 Influence of Patient Age on Implant Survival and Implant Success

**FIGURE 4:**
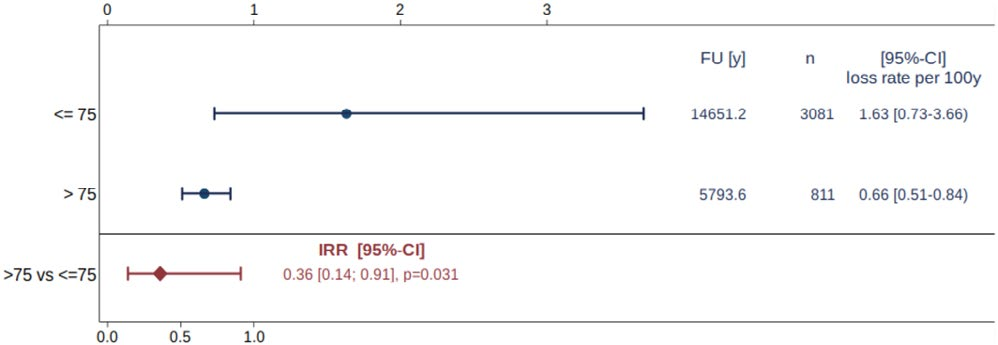
Meta‐analysis of implant loss rates comparing elderly the elderly (> 75 years), and younger study cohorts (≤ 75 years).

**FIGURE 5:**
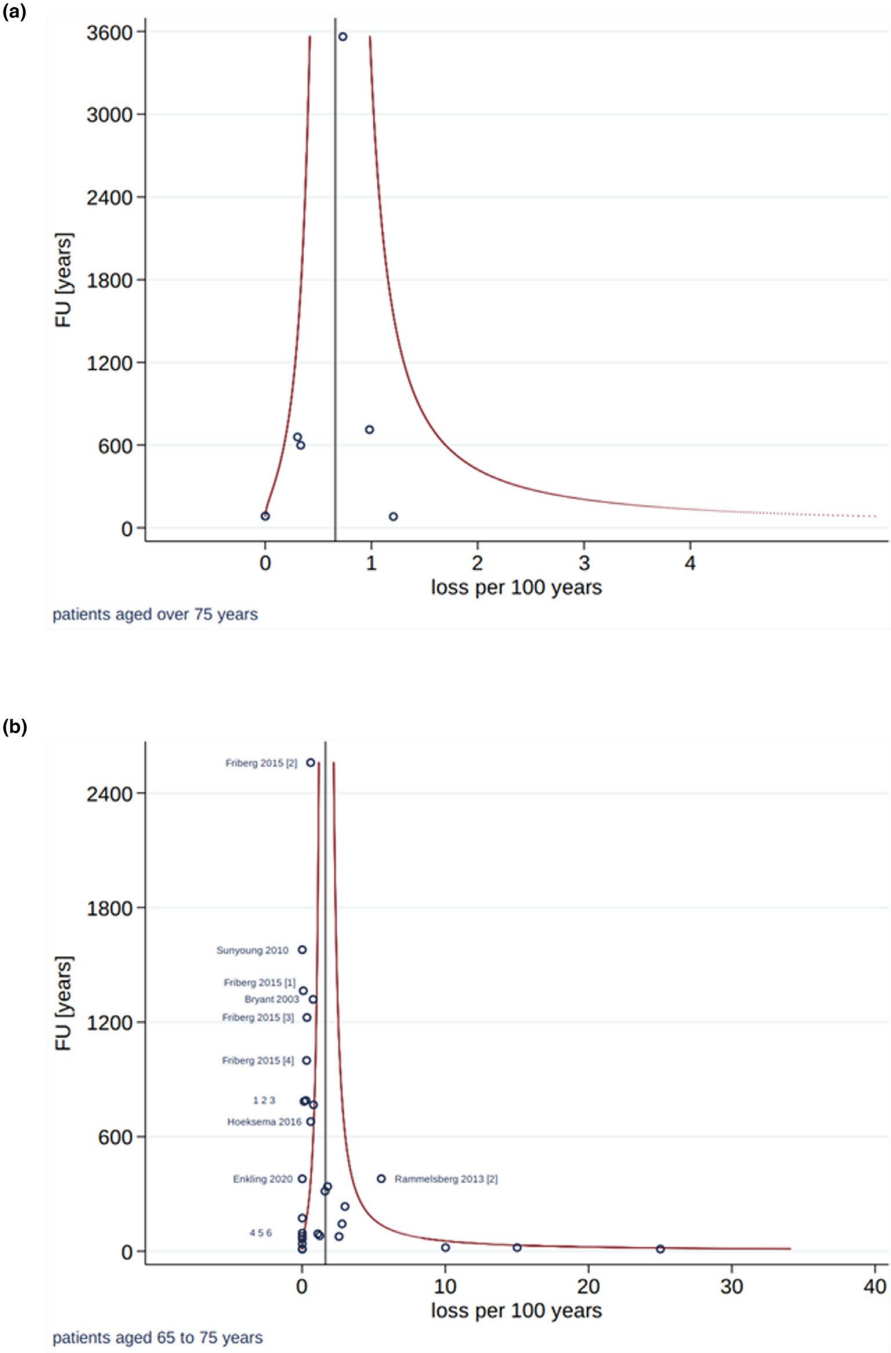
Funnel plots of included studies on implant loss in the elderly (> 75 years; a), and younger study cohorts (≤ 75 years; b).

**FIGURE 6:**
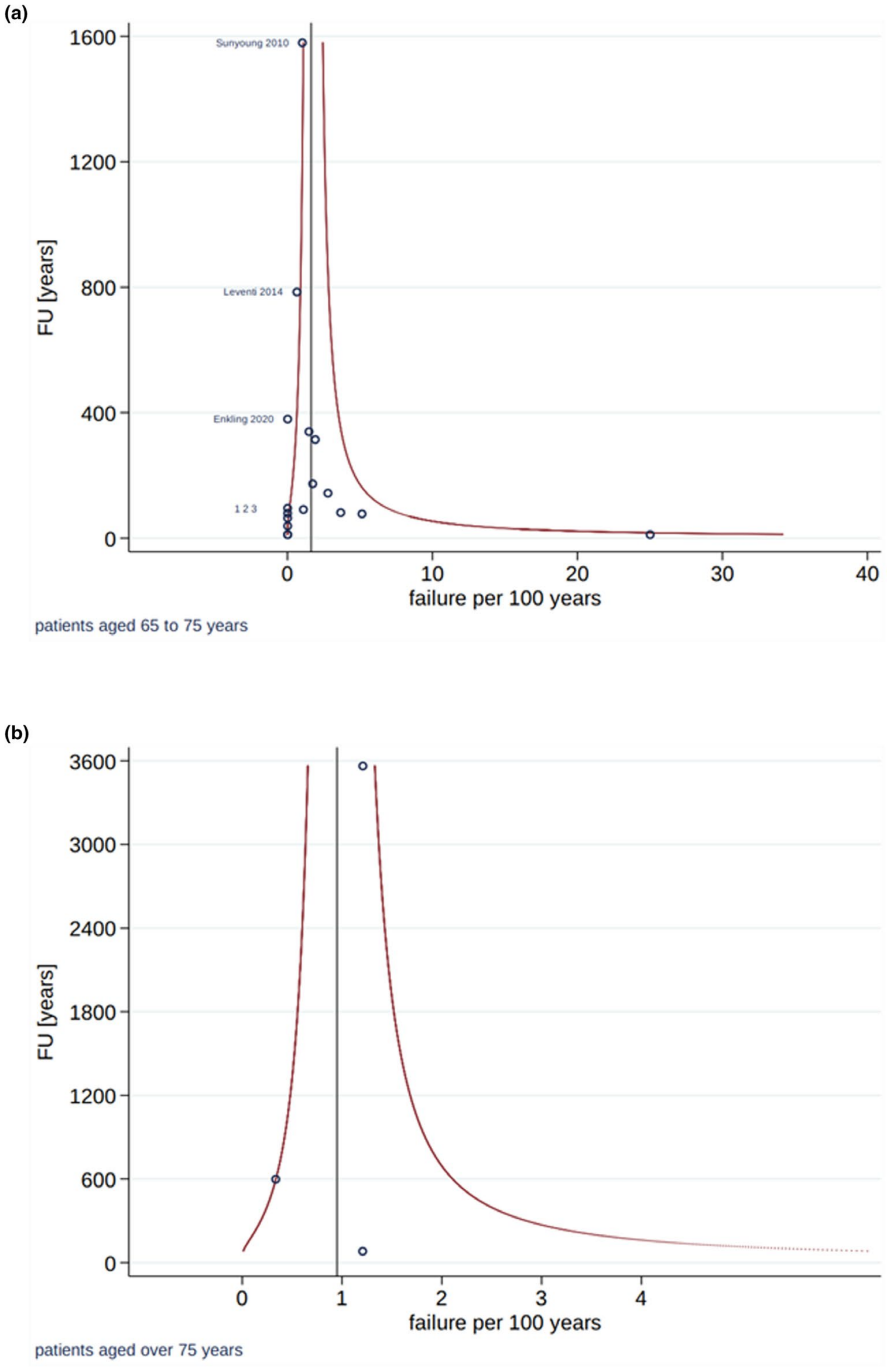
Funnel plots of included studies on implant success in the elderly (> 75 years; a), and younger study cohorts (≤ 75 years; b).

The estimated 5‐year survival rates were 92.1% in the younger and 96.8% in the older age group. A significant difference in implant survival comparing the two age groups was demonstrated (*p* = 0.031), with a 2.8‐fold lower loss rate in patients older than 75 years (Table 3; Figure 4). The estimated 5‐year success rates were 91.9% in the younger and 95.3% in the older age group, without a statistically significant difference between the groups (*p* = 0.229; Table 3). The heterogeneity among the included studies was small, considering the variance of exponentiated random effects (Table 3). The funnel plots did not indicate publication bias in terms of implant survival (Figure 5a,b) or implant success (Figure 6a,b).

**TABLE 3:** Meta‐analyses of implant survival and implant success.

		No. of studies	No. of impl. atFU	Exp. time (years)	Survival	Loss	Estimated survival rate (95% CI)*		Estimated loss rate per 100 implants (95% CI)*	*p* **	Var (95% CI)***
							3 year	5 year		IRR (95% CI)	
Implant survival											
Age	≤ 75	29	3081	14651.2	2980	101	95.2 (89.4–97.8)	92.1 (83.0–96.4)	1.63 (0.73–3.66)	0.031	1.87 (0.93–3.76)
	> 75	7	811	5793.6	773	38	98.0 (97.5–98.5)	96.8 (95.9–97.5)	0.66 (0.51–0.84)	0.36 [0.14; 0.91]	0.00 (0.00–0.00)
Implant success											
Age	≤ 75	17	1121	4287.0	1071	50	95.1 (89.5–97.7)	91.9 (83.1–96.2)	1.67 (0.77–3.64)	0.229	0.84 (0.09–7.77)
	> 75	3	329	4247.0	283	46	97.2 (91.2–99.1)	95.3 (85.7–98.5)	0.95 (0.30–3.03)	0.56 [0.22; 1.44]	0.09 (0.00–3963.69)

Abbreviations: FU, follow‐up; IRR, incidence rate ratio.

* Random‐effects Poisson regression for subgroups with two or more studies, Poisson regression for subgroups with one study.

** Random‐effects Poisson, global *p*‐value for more than two subgroups.

*** Variance of exponentiated random effects.

### 4.2 Subgroup Analyses Implant Survival

The effects of the jaw (upper/lower), the restoration type, and the type of retention (only in implant overdentures) on implant survival were analyzed independently of the age groups. Comparing studies, focusing on implant survival rates only in the mandible to both jaws, no difference could be demonstrated (*p* = 0.316) (Table 4). Unfortunately, no studies focusing on implant survival rates in the maxillae of patients older than 65 years could be identified. The restoration type had a significant influence on implant survival rates (global *p* < 0.001). Implants in implant‐assisted removable partial dentures showed a 6‐fold higher loss rate compared to implants in fixed restorations (*p* = 0.008). Other pairwise comparisons of restoration types showed no significant differences. The type of retention in implant overdentures had a significant effect on implant survival (global *p* = 0.002). Pairwise comparisons demonstrated a 5.6‐fold higher loss rate of implants with a single attachment compared to splinted attachments (*p* = 0.035). Furthermore, the loss rate in studies that reported combined survival rates of implants with splinted and single attachments was 3.7‐fold higher than in studies focusing exclusively on splinted implant restorations (*p* = 0.001). The heterogeneity among the included studies was small, considering the variance of exponentiated random effects (Table 4).

**TABLE 4:** Subgroup analyses of the effect of prosthetic parameters on implant survival, independent of age groups.

Effect of jaw, restoration, and retention type											
		No. of studies	No. of impl. at FU	Exp. time (years)	Survival	Loss	Estimated survival rate (95% CI)*		Estimated loss rate per 100 implants (95% CI)*	*p* ** IRR (95% CI)	Var (95% CI)***
							3 year	5 year			
Jaw	Mandible	27	2684	13153.8	2622	62	96.6 (85.8–99.2)	94.4 (77.5–98.7)	1.15 (0.27–4.98)	0.316	1.88 (0.48–7.36)
	Both	8	1106	6505.6	1030	76	94.4 (89.3–97.1)	90.8 (82.8–95.2)	1.91 (0.99–3.70)	2.04 [0.51; 8.27]	0.52 (0.28–0.97)
Restoration	FC	5	1374	6294.0	1347	27	98.4 (93.5–99.6)	97.3 (89.4–99.3)	0.55 (0.14–2.22)		0.62 (0.05–7.03)
	RP	1	87	234.9	80	7	91.3 (82.7–96.4)	86.0 (72.8–94.2)	2.98 (1.20–6.14)		—
	OD	26	1913	11733.9	1826	87	95.1 (86.9–98.2)	91.9 (79.1–97.0)	1.67 (0.61–4.58)		2.27 (0.99–5.21)
	Mixed	4	518	2182.0	500	18	97.5 (97.1–97.9)	95.9 (95.2–96.6)	0.82 (0.70–0.98)	< 0.001 (global)	0.00 (0.00–0.00)
									RP vs. FC	6.03 [1.59; 22.82], *p* = 0.008	
									OD vs. FC	2.30 [0.53; 9.99], *p* = 0.267	
									Mixed vs. FC	1.88 [0.83; 4.25], *p* = 0.130	
									OD vs. RP	0.54 [0.20; 1.49], *p* = 0.238	
									Mixed vs. RP	0.28 [0.23; 0.33], *p* < 0.001	
									OD vs. mixed	0.51 [0.17; 1.54], *p* = 0.230	
Retention	Single	15	891	3142.4	869	22	92.0 (73.3–97.7)	87.1 (59.6–96.2)	2.74 (0.76–9.82)		2.52 (1.11–5.75)
	Splinted	8	711	7085.4	674	37	98.6 (97.0–99.4)	97.7 (95.0–98.9)	0.47 (0.22–1.01)		1.23 (0.13–11.67)
	Mixed	1	68	340.0	62	6	94.8 (88.9–98.1)	91.5 (82.2–96.8)	1.76 (0.65–3.84)	0.002 (global)	—
									Splinted vs. single	0.18 [0.04; 0.89], *p* = 0.035	
									Mixed vs. single	0.68 [0.17; 2.65], *p* = 0.577	
									Mixed vs. splinted	3.73 [1.76; 7.87], *p* = 0.001	

Abbreviations: FU, follow‐up; IRR, incidence rate ratio.

* Random‐effects Poisson regression for subgroups with two or more studies, Poisson regression for subgroups with one study.

** Random‐effects Poisson, global *p*‐value for more than two subgroups.

*** Variance of exponentiated random effects.

### 4.3 Subgroup Analyses Peri‐Implant Parameters

The effect of patient age on BOP‐ and plaque‐positive implants, as well as on peri‐implant marginal bone‐level alterations was analyzed. Six study cohorts and 9 study cohorts reporting bleeding on probing and plaque levels, respectively, were identified. Patients aged 65–75 years showed significantly lower BOP‐ and (2‐fold) plaque‐positive implants (2.6‐fold), compared to patients older than 75 years (Table 5). Nine studies reporting marginal bone level alterations were identified (Table 6). The mean marginal bone level alterations in the older were 0.54 mm (95% CI: 0.14–0.94), and 0.95 mm (95% CI: 0.57–1.32) in the younger patient group, with no statistically significant difference between the groups (estimated mean difference: 0.41 mm; *p* = 0.189).

**TABLE 5:** Descriptive data on peri‐implant parameters.

Descriptive bleeding on probing (BOP) data							
Study	Age	Jaw	Type of restor	Retention	Mean FU [y]	#Impl. at FU	#BOP positive
Al Nawas et al. (2012)	≤ 75	Mandible	OD	Single	1.0	82	34
Al Nawas et al. (2012)	≤ 75	Mandible	OD	Single	1.0	78	32
Chow et al. (2017)	> 75	Mandible	OD	Single	5.2	126	62
Enkling et al. (2020)	≤ 75	Mandible	OD	Single	5.0	76	7
Huber et al. (2012)	≤ 75	Both	Mixed		1.0	65	8
Di Torresanto et al. (2014)	≤ 75	Mandible	OD	Single	2.0	40	7

Descriptive plaque data							
Study	Age	Jaw	Type of restor	Retention	Mean FU [y]	#Impl. at FU	#Plaque positive
Al Nawas et al. (2012)	≤ 75	Mandible	OD	Single	1.0	82	35
Al Nawas et al. (2012)	≤ 75	Mandible	OD	Single	1.0	78	36
Bakker et al. (2019)	> 75	Mandible	OD	Splinted	20.0	30	30
Chow et al. (2017)	> 75	Mandible	OD	Single	5.2	126	60
Mericske‐Stern and Zarb (1993)	≤ 75	Mandible	OD	Splinted	5.0	63	11
Mericske‐Stern and Zarb (1993)	≤ 75	Mandible	OD	Mixed	5.0	68	12
Schuster et al. (2020)	≤ 75	Mandible	OD	Single	1.0	20	4
Schuster et al. (2020)	≤ 75	Mandible	OD	Single	1.0	20	4
Di Torresanto et al. (2014)	≤ 75	Mandible	OD	Single	2.0	40	7

Abbreviations: FC, fixed complete; FU, follow‐up; OD, overdenture; RP, removable partial.

**TABLE 6:** Descriptive data on marginal bone level alterations.

Descriptive marginal bone level alterations (MBL)						
	Age group	Mean age	Mean FU [y]	#Patients at FU	Mean MBL	SD MBL
Leventi et al. (2014)	≤ 75	65.4	7.7	41	1.23	1.10
Enkling et al. (2022)	≤ 75	66.1	3	29	0.96	0.89
Enkling et al. (2020)	≤ 75	66.5	5	19	1.18	0.79
Hoeksema et al. (2016)	≤ 75	68	10	34	1.20	1.20
Ma et al. (2010)	≤ 75	72	10	79	0.29	0.53
Chow et al. (2017)	> 75	76.7	5.24	63	0.65	0.57
Jemt 1993	> 75	82.7	3	46	0.40	0.30
Bakker et al. (2019)	> 75	85.5	20	15	1.14	0.85
Becker et al. (2016)	> 75	89.4	1	31	0.10	0.10

## 5 Discussion

### 5.1 Statement of Principal Findings

This systematic review and meta‐analysis showed a statistically significant 2.8‐fold lower implant loss rate in patients over 75 years old compared to younger cohorts, with comparable 5‐year success rates. No specific studies on maxillary implant survival in patients over 65 years were identified. Implant survival was notably influenced by the type of restoration, with removable partial dentures showing a 6‐fold higher loss rate than fixed restorations. Similarly, single attachments in overdentures exhibited a 5.6‐fold higher loss rate compared to splinted attachments. Patients aged 65–75 years had significantly lower proportions of BOP‐positive (2‐fold) and plaque‐positive implants (2.6‐fold) than those over 75 years, though mean peri‐implant bone level changes were higher but not yet significant.

### 5.2 Strengths and Weaknesses of the Study

A notable strength of this study is the utilization of an established and well‐documented search protocol, which was further refined for this meta‐analysis. The protocol, previously employed in a systematic review on the effects of advanced age and systemic medical conditions on implant survival, ensured a high level of methodological rigor and reproducibility (Schimmel et al. 2018).

However, several limitations should be acknowledged. The restriction to studies published in English and German may have introduced inclusion bias, potentially overlooking valuable data from regions with significant expertise in geriatric implantology, such as the Scandinavian countries and Japan. This language limitation could result in an incomplete representation of global research findings.

Additionally, the absence of studies utilizing the International Delphi Consensus for Outcome Measures (ID‐COSM) (Tonetti et al. 2023) posed challenges for direct comparisons and may have restricted the scope of the analysis to conventional outcome measures. This highlights the need for broader adoption of standardized reporting frameworks in future studies.

Another limitation is the lack of detailed information on comorbidities within the geriatric study populations. Given the high prevalence of systemic conditions and polypharmacy in older adults (Anliker et al. 2023), the absence of these parameters may overlook important contributors to implant survival and success. Specifically, the potential influence of medications affecting bone metabolism, such as bisphosphonates or denosumab, was not assessed. An earlier review by Schimmel et al. (2018) highlighted the potential impact of systemic medical conditions on implant outcomes. In contrast, the present analysis did not explicitly evaluate systemic conditions or comorbidities, focusing instead on implant survival and peri‐implant health. Since the included studies did not consistently report systemic health data, it remains unclear to what extent multimorbid patients were represented in the study populations. Therefore, the findings of the present study should be interpreted with appropriate consideration. Recent literature suggests that systemic diseases and medications can influence dental implant outcomes, underscoring the importance of considering these factors in future research (D'Ambrosio et al. 2023). Furthermore, this review did not assess patient‐centered outcomes, such as oral health‐related quality of life (OHRQoL) or orofacial function, which are critical for understanding the broader impact of implant treatments. These aspects, however, have been thoroughly addressed in the recent ITI Consensus Conference (Abou‐Ayash et al. 2023; Schimmel et al. 2023).

Systematic reviews offer a comprehensive synthesis of existing literature, providing valuable insights into clinical questions. They employ predefined criteria for study inclusion and seek to extract consistent information from each study, often including formal appraisals of methodological quality (Cook 2019). This approach enhances the reliability and applicability of the findings. Although university settings may limit generalizability, they ensure standardized conditions, controlled patient selection, and structured follow‐up, improving data reliability. However, challenges arise when dealing with underreported conditions such as implant therapy in geriatric patients. The limited availability of high‐quality studies can compromise the robustness of conclusions.

### 5.3 Strengths and Weaknesses in Relation to Other Studies, Discussing Particularly Any Differences in Results

This review is the first to evaluate plaque accumulation and BOP alongside mean peri‐implant bone level changes in relation to patient age. By including these biological parameters, it provides a more comprehensive view of peri‐implant health than previous reviews, offering valuable insights into the factors influencing implant success in geriatric patients.

Compared to the earlier systematic review by Schimmel et al. (2018), which focused on the effects of advanced age and systemic medical conditions on implant survival, this review takes a broader approach by extending the analysis to include peri‐implant health indicators. Both reviews demonstrate that advanced age is not a contraindication for implant therapy, with high survival rates observed across age groups. However, the current review identifies clear survival advantages for splinted implants in overdentures and fixed restorations, providing stronger evidence for this specific treatment modality in older patients. This result offers a clear direction for improving long‐term outcomes in geriatric patients. In contrast, earlier reviews did not find such clear statistical results in regard to implant splinting (Schimmel et al. 2018; Kern et al. 2016), or on prosthetic factors or the retention types in overdentures, which adds unique value to the current study. However, splinting implants might also negatively influence the capacity to perform oral hygiene in patients with limited vision and dexterity. Hence, the lower cervical and success rates observed in simpler restorations, such as implant overdentures retained on unsplinted implants, may initially seem counterintuitive, especially given the need for uncomplicated hygiene procedures in older adults. However, it can be hypothesized that this finding can be explained by patients’ selections factors. In clinical practice, simpler prosthetic solutions are often chosen for older adults with poorer overall health status, reduced manual dexterity, or other limitations that may affect both implant cervical and oral hygiene maintenance. Consequently, these implant patients may already present with a higher risk profile, which could contribute to the observed outcomes rather that the prosthetic design itself being an independent risk factor.

Another strength of the current review is the inclusion of 27 studies encompassing approximately 3800 implants, representing, to the best of our knowledge, the largest evidence base on this topic to date. This robust sample size enhances the statistical power of the findings, enabling more definitive conclusions about the influence of age and restoration types on implant outcomes. Additionally, due to the increase in included studies, the funnel plots do not indicate any suspicion of publication bias. Although some of the studies, particularly those involving older patient cohorts, fall below the 95% confidence intervals, it is noticeable that these studies mainly involve longer follow‐up periods. The fact that these studies are slightly below the funnel is more likely due to the statistical prevalence of implant losses being higher in the first year compared to subsequent years (Lemmerman and Lemmerman 2005). However, since a relatively large number of studies with short follow‐up periods were included in this systematic review, this has most likely caused a shift in the funnel. Data has to be interpreted cautiously due to the short follow‐up period, which may not fully capture late complications and could lead to an overestimation of implant survival. Furthermore, the statistical analysis assumes that event occurrences follow a Poisson distribution, implying a constant hazard over time. While it is indeed likely that most events occur within the first year, the temporal distribution of events may vary across studies and subgroups. This simplification may introduce some degree of deviation from real‐world dynamics. Owing to the difficulty in extracting the exact age of each individual patient and the associated outcomes from the included studies, the mean age was used as the criterion for study inclusion and exclusion. This approach most likely resulted in the inclusion of studies in which some individual patients did not fully meet the age‐related inclusion criteria.

### 5.4 Unanswered Questions and Future Research

A critical area for future investigation lies in the observed reduction in marginal bone level alterations despite the presence of high BOP values among elderly patients, as also demonstrated in studies from our group (Ramseier et al. 2021; Enkling et al. 2020). The underlying biological mechanisms driving this phenomenon remain unclear but may be linked to immunosenescence, a term describing age‐related declines in immune system function (Ebersole et al. 2016). While the lower BOP values in elderly patients might superficially suggest improved peri‐implant or periodontal health, it is likely that these findings reflect diminished inflammatory responses rather than the absence of disease activity. This interpretation is supported by Müller et al. (Muller et al. 2022), who highlighted that periodontitis and peri‐implantitis in elderly institutionalized patients often present with atypical clinical signs, such as minimal bleeding despite ongoing tissue destruction.

Further, experimental studies such as those by Meyer et al. (2019) demonstrate that elderly individuals may experience altered clinical and microbiological responses to inflammation. In their investigation of mucositis and gingivitis in individuals aged 70 and older, microbial colonization was similar to that observed in younger cohorts; however, the clinical expression of inflammation was notably muted. This reduced clinical reactivity raises concerns about underdiagnosis or delayed diagnosis of peri‐implant diseases in aging populations.

The systematic review by Preshaw et al. (2017) further emphasizes that immunosenescence affects multiple immune pathways, including T‐cell function, neutrophil activity, and cytokine production. This “silent inflammatory progression” highlights the need for clinicians to adopt alternative diagnostic approaches, such as radiographic assessment or biomarkers of tissue destruction, to accurately monitor peri‐implant health in elderly patients.

Given the possibly important clinical significance of immunosenescence, future studies should focus on exploring its role in peri‐implant health and disease progression in aging populations. Additionally, research is needed to identify alternative, age‐appropriate diagnostic criteria that account for reduced inflammatory responses in elderly patients.

Finally, integrating a standard set for systemic health parameters, such as comorbidities, polypharmacy, and nutritional status, and dedicated implant parameters into future studies could provide a more comprehensive understanding of the multifactorial nature of improvement in function, implant success, and peri‐implant health in geriatric patients and facilitate further aggregation of results in meta‐analyses (Tonetti et al. 2023).

## 6 Conclusion

Although not subject of the current systematic review and meta‐analysis, implant therapy in old and very old adults should only be considered after careful evaluation of the medical risks, the possible benefits, and the individual capacity for oral hygiene and access to care in order to achieve benefit, not harm, to a geriatric patient close to dependency.

Within the limitations of the systematic review and meta‐analysis, current data indicates that patients older than 75 years have higher or similar 5‐year implant survival and success rates compared to those aged 65–75 years. Although older patients demonstrated higher frequencies of plaque and bleeding on probing, these did not correlate with significant differences in peri‐implant bone‐level changes. Splinting implants in overdentures results in 5.6 times higher implant survival rates.

## Author Contributions

**Samir Abou‐Ayash:** methodology, conceptualization, writing – original draft, data curation. **Monika Bjelopavlovic:** methodology, validation, writing – review and editing, writing – original draft, software. **Pedro Molinero‐Mourelle:** writing – review and editing, formal analysis, methodology, data curation, visualization. **Martin Schimmel:** supervision, resources, project administration, funding acquisition, investigation.

## Ethics Statement

The authors have nothing to report.

## Conflicts of Interest

The authors declare no conflicts of interest.

## Data Availability

The data that support the findings of this study are available from the corresponding author upon reasonable request.

## Appendix 1

**Search Terms**

*Medline (via PubMed) and PubMed Central*

(elder*[Title/Abstract] OR senior*[Title/Abstract] OR “advanced age”[Title/Abstract] OR “65 years” [Title/Abstract] OR “older” [Title/Abstract])

AND

(“Dental Implants”[Mesh] OR “Dental Implants, Single Tooth”[Mesh] OR “Dental Prosthesis, Implant‐Supported”[Mesh] OR “Denture, Overlay”[Mesh] OR Overdenture[Title/Abstract] OR “attachment type” [Title/Abstract] OR “Dental Abutments”[Mesh] OR “Alveolar Ridge Augmentation”[Mesh])

AND

(“Middle Aged”[Mesh] OR “Adult”[Mesh] OR “Adult”[Title/Abstract] OR young*[Title/Abstract])

AND

(“quality of life” [Title/Abstract] OR survival[Title/Abstract] OR “Mastication”[Mesh] OR “Patient Satisfaction”[Mesh] OR “Patient Outcome Assessment”[Mesh] OR “Patient Reported Outcome Measures”[Mesh] OR “Treatment Outcome”[Mesh] OR “Peri‐Implantitis”[Mesh] OR “Oral Health”[Mesh] OR complication[Title/Abstract] OR success[Title/Abstract] OR “implant failure*”[Title/Abstract] OR “bone level”[Title/Abstract] OR “bone loss”[Title/Abstract] OR “static”[Title/Abstract] OR “dynamic”[Title/Abstract] OR “guided”[Title/Abstract] OR “Drug‐Related Side Effects and Adverse Reactions”[Mesh] OR “Surgery, Computer‐Assisted”[Mesh])

*Cochrane Library*

(elder*:ti,ab,kw OR senior*:ti,ab,kw OR “advanced age”:ti,ab,kw OR “65 years”:ti,ab,kw OR older:ti,ab,kw)

AND

([mh “Dental Implants”] OR [mh “Dental Implants, Single Tooth”] OR [mh “Dental Prosthesis, Implant‐Supported”] OR [mh “Denture, Overlay”] OR Overdenture:ti,ab,kw OR “attachment type”:ti,ab,kw OR [mh “Dental Abutments”] OR [mh “Alveolar Ridge Augmentation”])

AND

([mh “Middle Aged”] OR [mh “Adult”] OR Adult:ti,ab,kw OR young*:ti,ab,kw)

AND

(“quality of life”:ti,ab,kw OR survival:ti,ab,kw OR [mh “Mastication”] OR [mh “Patient Satisfaction”] OR [mh “Patient Outcome Assessment”] OR [mh “Patient Reported Outcome Measures”] OR [mh “Treatment Outcome”] OR [mh “Peri‐Implantitis”] OR [mh “Oral Health”] OR complication:ti,ab,kw OR success:ti,ab,kw OR “implant failure*”:ti,ab,kw OR “bone level”:ti,ab,kw OR “bone loss”:ti,ab,kw OR static:ti,ab,kw OR dynamic:ti,ab,kw OR guided:ti,ab,kw OR [mh “Drug‐Related Side Effects and Adverse Reactions”] OR [mh “Surgery, Computer‐Assisted”])

## Appendix 2

**Table:**

Author and year	Reason for exclusion	Title
Esposito et al. (2017)	Zygomatic implants	Conventional drills vs. piezoelectric surgery preparation for placement of four immediately loaded zygomatic oncology implants in edentulous maxillae: results from 1‐year split‐mouth randomised controlled trial
Schwarz et al. (2014)	Mean age younger than 65 at the baseline	Early Loading of Implants with Fixed Dental Prostheses in Edentulous Mandibles: 7.2‐Year clinical results from a prospective study
Van Doorne et al. (2023)	Mean age younger than 65 at the baseline	Five years clinical outcome of maxillary mini dental implant overdenture treatment: A prospective multicenter clinical cohort study
Jung et al. (2013)	Mean age younger than 65 at the baseline	A prospective, controlled clinical trial evaluating the clinical radiological and aesthetic outcome after 5 years of immediately placed implants in sock‐ ets exhibiting periapical pathology
Marrone et al. (2013)	Mean age younger than 65 at the baseline	Prevalence and risk factors for peri‐ implant disease in Belgian adults
Ravald et al. (2013)	Data reporting unclear/data could not be extracted	Long‐term evaluation of Astra Tech and Bra °nemark implants in patients treated with full‐arch bridges. Results after 12–15 years
van Eekeren et al. (2016)	Mean age younger than 65 at the base line	Crestal bone changes in macrogeomet‐ rically similar implants with the implant–abutment connection at the crestal bone level or 2.5 mm above: a prospective randomized clinical trial
Mericske‐Stern et al. (1994)	Data reporting unclear/data could not be extracted	Peri‐implant mucosal aspects of ITI implants supporting overdentures
Grossmann et al. (2007)	No implants reported	Treatment with double crown–retained removable partial dentures and oral health–related quality of life in middle‐ and high‐aged patients
Merli et al. (2012)	Mean age younger than 65 at the baseline	Immediate versus early non‐occlusal loading of dental implants placed flapless in partially edentulous patients: A 3‐year randomized clinical trial
Muller et al. (2013)	Duplicate cohort	Implant‐supported mandibular overdentures in very old adults: a randomized controlled trial
Berretin‐Felix et al. (2009)	Mean age younger than 65 at the baseline	Effects of mandibular fixed implant‐supported prostheses on masticatory and swallowing functions in completely edentulous elderly individuals
Brocard et al. (2000)	Mean age younger than 65 at the baseline	A Multicenter report on 1022 consecutively placed iti implants: a 7‐year longitudinal study
Brugger et al. (2015)	Mean age younger than 65 at the baseline	Implant therapy in a surgical specialty clinic: an analysis of patients, indications, surgical procedures, risk factors, and early failures
Compton et al. (2017)	Mean age younger than 65 at the baseline	Dental implants in the elderly population: a long‐term follow‐up
Schuster et al. (2019)	Mean age younger than 65 at the baseline	Influence of age and time since edentulism on masticatory function and quality of life in implant‐retained mandibular overdenture wearers: 1‐year results from a paired clinical study
Michaud et al. (2012)	Follow‐up less than 1 year	Measuring patient‐based outcomes: Is treatment satisfaction associated with oral health‐related quality of life?
Pan et al. (2010)	Follow‐up less than 1 year	Does mandibular edentulous bone height affect prosthetic treatment success?
Preciado et al. (2012)	Data reporting unclear/data could not be extracted	Differences in impact of patient and prosthetic characteristics on oral health‐related quality of life among implant‐retained overdenture wearers
Landa et al. (2001)	Data reporting unclear/data could not be extracted	A prospective 2‐year clinical evaluation of overdentures attached to nonsplinted implants utilizing ERA attachments
Heydecke et al. (2008)	Follow‐up less than 1 year	Do mandibular implant overdentures and conventional complete dentures meet the expectations of edentulous patients?
Olerud et al. (2012)	Data reporting unclear/data could not be extracted	Oral status, oral hygiene, and patient satisfaction in the elderly with dental implants dependent on substantial needs of care for daily living
Trezubov et al. (2018)	No survival/complications	Clinical substantiation of the sparing and less invasive implant prosthetics of the edentulous lower jaw method
Cummings and Arbree (1995)	Data reporting unclear/data could not be extracted	Prosthodontic treatment of patients receiving implants by predoctoral students: five‐year follow‐up with the IMZ system
Cordioli et al. (1997)	Data reporting unclear/data could not be extracted	Mandibular overdentures anchored to single implants: A five‐year prospective study
Komagamine et al. (2023)	No survival/complications	The effect of single‐implant overdentures on cognitive function in older adults: a 3‐year follow‐up report
Heydecke et al. (2005)	No survival/complications	Cost‐effectiveness of mandibular two‐implant overdentures and conventional dentures in the edentulous elderly
Geckili et al. (2011)	Follow‐up less than 1 year	Impact of mandibular two‐implant retained overdentures on life quality in a group of elderly Turkish edentulous patients
Visser et al. (2016)	Mean age younger than 65 at the baseline	A 15‐year comparative prospective study ofsurgical and prosthetic care and aftercare of overdenture treatment in the atrophied mandible: augmentation vs. nonaugmentation
Naert et al. (1997)	Mean age younger than 65 at the baseline	The reliability of implant‐retained hinging overdentures for the fully edentulous mandible. An up to 9‐year longitudinal study
Kim et al. (2013)	Mean age younger than 65 at the baseline	Clinical use of alumina‐toughened zirconia abutments for implant‐ supported restoration: prospective cohort study of survival analysis
Zhang et al. (2014)	No survival/complications	A new classification of peri‐implantbone morphology: a radiographic studyof patients with lower implant‐supported mandibular overdentures
Martin‐Ares et al. (2016)	No implant survival	Prosthetic hygiene and functional efficacy in completely edentulous patients: satisfaction and quality of life during a 5‐year follow‐up
Enkling et al. (2017)	Duplicate cohort	Chewing efficiency, bite force and oral health‐related quality of life with narrow diameter implants—a prospective clinical study: results after 1 year
Enkling et al. (2019)	No implant survival	A prospective cohort study on survival and success of one‐piece mini‐implants with associated changes in oral function: Five‐year outcomes
Cannizzaro et al. (2017)	Mean age younger than 65 at the baseline	Immediate loading of two (fixed‐on‐2) vs. four (fixed‐on‐4) implants placed with a flapless technique supporting mandibular cross‐arch fixed prostheses: 3‐year results from a pilot randomized controlled trial
Kuoppala et al. (2012)	Mean age younger than 65 at the baseline	Outcome of implant‐supported overdenture treatment—asurvey of 58 patients
Negri et al. (2014)	Mean age younger than 65 at the baseline	The effect of age, gender, and insertion site onmarginal bone loss around endosseous implants: results froma 3‐year trial with premium implant system
Ochi et al. (1994)	Follow‐up less than 1 year	Patient demographics and implant survival at unicovering dental implant clinical research group interim report No. 6
Lowy et al. (2019)	Mean age younger than 65 at the baseline	The effect of platform‐switching plus laser grooving on peri‐implant hard and soft tissue level: a randomized, controlled, blinded clinical trial
Katheng et al. (2021)	Duplicate cohort	Masticatory performances and maximum occlusal forces of immediate and conventional loaded two‐implant supported overdentures retained by magnetic attachments: preliminary study of randomized controlled clinical trial
Roynesdal et al. (1998)	Duplicate cohort	A comparative clinical study of three different endosseous implants in edentulous mandible
Bryant and Zarb (1998)	Data reported in a mean follow‐up way	Osseointegration of oral implants in older and younger adult
Emami et al. (2010)	Only quality of life reported outcomes	Better oral health related quality of life: type of prosthesis or psychological robustness?
Pan et al. (2008)	Only quality of life reported outcomes	Sex differences in denture satisfaction
Sanchez‐Perez et al. (2021)	Data reporting unclear/data could not be extracted	Primary stability and PES/WES evaluation for immediate implants in the aesthetic zone: a pilot clinical double‐blind randomized study
Temmerman et al. 2017	Data reporting unclear/data could not be extracted	An open, prospective, non‐randomized, controlled, multicentre study to evaluate the clinical outcome of implant treatment in women over 60 years of age with osteoporosis/osteopenia: 1‐year results
